# Life

**Published:** 1873-01

**Authors:** 


### LIFE.

DEAR DOCTOR:—Your note of yesterday ordering me to "write an article for the Bistoury and mail it to you at once," only gives me a little over forty minutes in which to select a subject and write it up for one of the sharpest journals of the day, to be read and criticised all over the land. When articles are to be furnished upon short notice, like this, it would be well to suggest a subject for the writer; but the Editor of the Bistoury is a curious genius, and thinks, doubtless, that every one can write as rapidly as he. Well, life is all curious; it is a curious world we live in, and curious folks we live with; and this idea shall form the basis of my remarks this time.

Human life as we study it, becomes an increasing wonder every moment:—From the first pulsation of the heart, to the last sigh of agony and look of despair. If we gaze upon the blanched cheek, the marble forehead, the fixed eye, the still lip and inexpressive face, we call it death, but it is not; for the qualities we admired, the thoughts we prized, the character we loved, the mind we revered, the soul that we felt was anchored fast to ours, has only just begun to live. If in our fancy, we attempt to follow it in its new abode, we are lost in a moment, in the vast unmeasured, boundless, unexplored and wondrous realms, if infinity, which lie so far beyond us that the wildest imagination cannot attempt to portray its beginning, and yet, so near us, that while we hear the echo of voices that we loved, the *loved one* may be standing in the blazing sunlight of that better land. I will not pursue this thought further, for I have no desire to lead or to be led into the inextricable labyrinth of metaphysics.

Life's history is but little less curious than life itself ; and to him who calmly and considerately reviews his own career, it seems only like a passing panorama, and he is unable to realize that in the pictured scenes before him, he was silly enough to have been the chief performer ; and yet, with the anxious throng, on he goes, taxing every energy to make his life a success,—never realizing how few of the busy millions win.

The man who is intoxicated with the sovereign idea of amassing a fortune, and does it at the expense of his personal comfort, or at the cost of other's happiness, with all his possessions, has made a magnificent failure, he has only stood upon the scales to weigh down real manhood, and when he has stepped off, the world around him breathes freer, and the balances have a chance of adjustment. He who lives to gratify his passions or appetite, kindles within himself a burning fire, which consumes every atom of Godlike nobility ; and when he has been removed, society feels happy to be liberated from human poison, and almost inclined to pity the next company he may come in contact with.

He who devotes himself to the achievement of position, for the honor it brings, may occupy high places in political life ; he may become an artful mouser, and hold office at the expense of principle ; but he makes a failure, and his hacks rejoice that death comes so kindly to relieve them of their load. He who earnestly desires to make life a sublime success, must investigate his own nature, and cultivate an intimate acquaintance with his own heart ; he must study the wants and necessities of those around him, and if providence has been prodigal of benefits to him, he must feel it to be his first duty and his highest honor to hold up and help others into the flood of fortune or to extend the beneficent hand to those below him ; and with feelings far too eloquent to be uttered, urge others on to the consummation of an exalted destiny.—Such men have lived, and still live ; some have perished for principle, some for humanity, some for country : but, whenever, or whereever they have fallen, mankind loves to look, linger, and weep. These men are like monuments, which have attained such altitudes that the sunlight always crowns their summits. No matter whether such men are followed to their tombs or not by the gorgeous array of pomp, wealth and fashion, their lives have been truly grand and their memories live and flourish green and beautiful in the grateful hearts they left behind them. It is perhaps one of the most singular features connected with life, that in looking *at* it, we form opinions and make resolves at which we are astonished when we come to look it *over*. When we look at life we determine that we will avoid the rough places, that in our voyage we will stear clear of difficulty and danger, and with bright prospects, and light hearts, we set sail with a clear sky above us, and gentle winds to waft us over the still, placid sea. We seem anxious to know all the obstacles which may lie before us ; but a wise and inscrutable Providence has kindly concealed them, and on we go, striking a rock *here*, meeting an iceberg there, and before we know it, the winds howl, the seas roar, and the storm rages. We trim our ship, adjust everything to meet the emergency, and ultimately find our way into the calm, smiling waters. We feel then, that we are conquerors ; thanks to the raging storm, but for it, we should never have known the joy of deliverance, or the ecstacy of triumph ! If our lives were exactly as we wished them to be, the journey would become a dull, tiresome, monotony and we would long for death, or anything, to deliver us from its tedious sameness. The mariner dreads the still, mirror-faced ocean, with its ceaseless and disgusting surge and roll ; so would we dread and despise a life, going on and on, in one endless round, a continuous dream, without change, animation, or expectancy. The traveler has no conciousness that he has glided ten thousand miles, over a smooth sea, but every storm he has encountered, every sand-bar he struck, every lighthouse that warned him, every perishing crew he saved, he can never forget ; and, with emotions of joy, he recounts the dangers he has passed, and the famishing ones he rescued from the sinking ship. So it is with life ; we have no conception or appreciation of the bright days we have passed, when all was quiet and undisturbed ; it only looks to us like a dream. He who measures life by the days he has lived, by the nights he has slumbered, or the viands he has consumed, has no more to boast of, than the beasts which perish. He who runs from encounters and fears conflicts, is a cravan coward ; but he who meets life in all its forms and phases, who dares dangers, who conquors enemies, subdues foes, and defends the right, is a brave man, and always wins. Let us never dread the storms or rough places in the voyage of life, if we desire to be immortalized as real heroes; but, let us remember that the circumstances we meet develops our characters, and give us a place in the heart and history of the world. We have inspiring examples, worthy of imitation, given to us in history, stretching through all the ages, from the present moment, to the day that the Great Reformer gave up his life for the good of a race who persecuted him. And, as the rocks were rending, the sun hiding his face, and devoted ones rising from their dusty beds, he exclaimed in accents that rung through Earth and Heaven, and the echo of which will smite the most distant shore of eternity, "it is finished"! Life was a sublime success,—an ignominious and shameful death a splendid victory. NED.

